# Meeting of the Medical Society of South Western New York

**Published:** 1857-09

**Authors:** 


*Meeting of the Medical Society of South Western New York.*—The members of the Medical Society of South Western New York, held their *third* quarterly session for the present year, at the society's rooms in Westcott's block, Jamestown, on the 5th Aug. The Society was called to order, at 1 o'clock, by the President, Dr. G. W. Hazeltine, and in the temporary absence of the Secretary, Dr. O. C. Gibbs was chosen secretary *pro tem.*

Several new members were admitted, giving evidence of a growing interest in this young and vigorous society.

Several medical and surgical cases of much interest were presented by Drs. Hazeltine, Axtell, Bemus, and Gibbs, which were examined by the other members present, and dicussed with much interest and spirit.

The standing committee on special diseases, asked permission to defer presenting their reports until the next meeting, with the exception of Dr. T. D. Strong, of Westfield, who presented a partial, though very interesting report upon the use of chloroform in obstetrics. Discussion upon this repert was deferred for want of time.

At 4 o'clock, the members present, their wives, and invited guests, sat down to a sumptuous dinner prepared by the Messrs. Westcott. A blessing was asked by the Rev. Mr. Norton, after which Doctors, Reverends, Esquires, &c., with their wives, discussed the excellent dinner with seemingly good relish. When the appetite of all was satisfied, the President, Dr. Hazeltine, made a short but appropriate speech, concluding with an excellent sentiment, to which Dr. Washburn responded in a very happy manner. Sentiments were read and responses made, until the shades of evening gave warning that it was time to adjourn preparatory to the evening exercises. The best feeling prevailed, and we are sure none regretted having been present on that occasion.

At 8 o'clock, P. M., the members of the society met in the Lecture Room of the Congregational Church, when A. Hazeltine, Esq., read a Biography of the late Dr. Laban Hazeltine, before a large and appreciative audience; after which the regular address was delivered by Dr. T. D. Strong, of Westfield.

The address was able and interesting, doing credit to the lecturer, as well as he to the subject. Dr. Strong discussed the merits of homœopathy with ability and candor, and whatever might have been the previous opinions of any of his hearers, they could not have failed to be interested in the discussion of a question of vital importance to all.

In the first place he quoted largely from the writings of Hannheman and other distinguished homœopaths, in order to show the foolish and visionary nature of its pretensions. He showed conclusively that the dynamic power, which medicines are claimed to acquire by *dilution* and trituration, is an arrant humbug. A drop of laudanum diluted with a barrel of water does not gain strength by the dilution, however much it may be shaken. Physicians have never discovered that paregoric acquired increased soporific powers, or a deadly potency, by being carried about on horseback for weeks previous to administration. In the second place, he showed the loose manner of homœopathic observation, and the unscientific and unreliable nature of the pretended facts upon which the system is based. He quoted from distinguished homœopathic authority to show that they disregard the sciences of anatomy and pathology altogether; treating symptoms only, regardless of the ultimate nature and character of the disease which give rise to such symptoms. In the third place, he introduced facts to show that where homœopathy had been put to the rigid test, under the observation of disinterested persons, as has repeatedly been done in European hospitals, it has, in every instance, failed to produce curative results, at all comparable with regular medicine. In some instances, the homœopaths making such trial have voluntarily retired in chagrin, or been compelled to relinquish their post by the proper authorities. He showed that the reputed success attending homœopathic treatment, in certain diseases, was explainable on the ground of their loose and unscrupulous manner of diagnosis. Thus, in Cincinnati two years ago, homœopaths reported remarkable success in the treatment of cholera, but when their report was investigated by the city Board of Health, it was found they had reported no cases of cholera morbus, diarrhœa, or dysentery: all diseases of a diarrhœaic character were classed with cholera, and all recoveries were reported as cures from that disease. In the fourth place, he showed that homœopaths themselves had but little confidence in their system, for, in urgent cases, they not unfrequently disregard their theory of increased power by dilution, and administer medicines in full doses.

We have not attempted a full synopsis of Dr. Strong's address, for space would not permit.

At the conclusion of the address, the members of the society returned to their rooms, and continued in session until past 11 o'clock, P. M. They passed resolutions to publish the biography of Dr. Laban Hazeltine, and to increase the utility of the society by lengthening the sessions.

The next meeting will be held at Fredonia, commencing at 10 o'clock, A. M., and continuing through two days.

The number present was unusually large, and the meeting was of the most cordial and fraternal character. The professional duties of the physician impress cares and responsibilities, rarely equalled by any other vocation in life, and it is of good omen that the physicians of South Western New York, are alive to those responsibilities. May the society and its members continue to prosper. G.

